# Screening Routine Clinical Notes for Epilepsy Surgery Candidates Using Large Language Models

**DOI:** 10.1002/acn3.70427

**Published:** 2026-05-05

**Authors:** Uriel Fennig, Nadav Amir, Maya Schiller, Roni Loebenstein, Johnatan Nissan, Marina Boxer, Shany Guly Gofrit, Gal Goshen, Sándor Beniczky, Nicola Maggio

**Affiliations:** ^1^ Department of Neurology The Sheba Medical Center at Tel Hashomer Ramat Gan Israel; ^2^ Dina Recanati School of Medicine Reichman University Herzliya Israel; ^3^ Sheba Medical Center at Tel Hashomer Ramat Gan Israel; ^4^ The Faculty of Medical and Health Sciences Tel Aviv University Tel Aviv Israel; ^5^ Department of Clinical Medicine University of Copenhagen Copenhagen Denmark; ^6^ Department of Clinical Neurophysiology Copenhagen University Hospital Copenhagen Denmark; ^7^ Danish Epilepsy Centre Dianalund Denmark; ^8^ Gray Faculty of Medical and Health Sciences – Department of Neurology and Neurosurgery Tel Aviv University Tel Aviv Israel

**Keywords:** decision support, epilepsy surgery, large language models, screening

## Abstract

**Objective:**

Epilepsy surgery is severely underutilized despite proven efficacy, with substantial under‐referral of eligible patients in routine clinical practice. This study evaluated the potential role of large language models (LLMs) as decision‐support tools for screening unstructured clinical notes to identify epilepsy surgery candidates and stratify them according to prognostic indicators.

**Methods:**

We retrospectively analyzed free‐text medical records in a non‐English language (Hebrew) from 110 patients in a tertiary epilepsy clinic. Six LLMs (Gemini 2.5 Pro, 2.5 Flash, 2.0 Flash; GPT‐5, GPT‐5 mini; and o4‐mini) were prompted to extract surgical eligibility criteria, parameters of the Seizure Freedom Scale (SFS) for surgical prognostication, completion of presurgical evaluations, and previous surgical consideration. Model outputs were compared with expert manual review.

**Results:**

Model performance in identifying core eligibility parameters demonstrated high sensitivity (up to 1.00) and specificity (up to 0.96), with favorable predictive values (PPV up to 0.92, NPV up to 1.00). Majority voting yielded near‐perfect sensitivity (1.00 in this cohort) for identifying surgical eligibility. Notably, 45% (13/29) of patients meeting surgical criteria had no prior consideration of surgery. Models demonstrated high accuracy in SFS score evaluation (sensitivity 0.95, specificity 0.93) and strong performance in identifying completed presurgical evaluations.

**Interpretation:**

These findings suggest the potential role of LLMs to act as decision‐support tools for identifying patients who may benefit from surgical evaluation but have not been recognized in routine care. This is supported by the models' high performance in correctly identifying eligible patients, as well as prognostic parameters. As this performance was achieved using off‐the‐shelf general‐purpose models applied directly to raw, non‐English clinical notes, it suggests a practical and scalable screening approach across diverse clinical settings.

## 1 Introduction

Epilepsy surgery is considerably under‐utilized despite proven efficacy, with an estimated 1% of surgical candidates being referred to a comprehensive epilepsy service for consideration of surgery [1, 2, 3]. Recent years have shown a trend towards increased awareness and reduced latency from drug‐resistant epilepsy (DRE) establishment to surgical workup; however, delays are still significant, averaging over 13 years before referral [4].

Although patients with drug‐resistant epilepsy should ideally be evaluated for surgical candidacy during routine clinical care, substantial under‐referral persists in practice. Consequently, additional mechanisms to support the identification of eligible patients may be beneficial. One such mechanism may be through automated decision‐support tools to assist clinicians in identifying eligible patients during routine care, or through systematic screening when needed. Previous works have demonstrated the ability of natural language processing (NLP), and more recently LLMs, to extract specific epilepsy‐related information from unstructured clinical text [5, 6, 7]. Notably, Fang et al. used LLMs to extract epilepsy and seizure types, current ASMs, and associated symptoms, with promising results [7].

While NLP methods specifically for the purpose of identifying surgical epilepsy candidates have also been developed [8, 9], these approaches face significant barriers to real‐world adoption. They rely on labor‐intensive feature engineering, supervised learning, and result in site‐ and language‐specific models. Adoption in differing medical sites, which are heterogeneous in documentation style or language, would not be possible without substantial retraining, creating an implementation barrier.

The advent of LLMs is transforming the field of NLP and offers new opportunities for analyzing unstructured clinical data [10]. Off‐the‐shelf LLMs can directly process raw, heterogeneous clinical narratives with minimal preprocessing, and with improved adaptability, lowering the barrier to widespread adoption [11]. Notably, recent developments have introduced domain‐specific medical LLMs, such as Med‐PaLM and related models, which are designed to support complex clinical reasoning through fine‐tuning on medical corpora [12, 13]. In parallel, state‐of‐the‐art general‐purpose LLMs remain widely used due to their accessibility, strong zero‐shot capabilities, and ability to process clinical information without task‐specific training.

We assessed the efficacy of such off‐the‐shelf LLMs using zero‐shot prompting to screen raw, unstructured clinical notes, flag epilepsy patients suitable for a surgical evaluation, and stratify them according to the likelihood of achieving seizure freedom. This practical and broadly available method has the potential to support early and effective identification of candidates for epilepsy surgery.

## 2 Materials & Methods

### 2.1 Data Collection, Setting, and Study Population

This retrospective study was conducted at the adult epilepsy clinic of Sheba Medical Center. We included clinical follow‐up notes from electronic health records (EHRs) of a consecutive series of 116 patients presenting to the clinic in reverse chronological order from February 2025 backwards.

In anticipation of a pilot study phase, to prevent data leakage, the study population was split temporally. The first 16 patients (the most recent cases) were designated as the pilot cohort. The subsequent 100 consecutive patients were designated as the validation cohort.

After review of the collected data, six patients were excluded from the study due to a lack of complete or usable visit summaries (clinical narratives of less than 200 words, and/or isolated follow‐up records without mention of basic clinical characteristics—all deemed as insufficient to represent accurate clinical states). This resulted in a validation cohort of 94 patients (excluding the 16 pilot‐study cases). The visit summaries for this cohort served as the primary data source for both the manual and AI‐based analyses. All raw clinical data were in the Hebrew language.

### 2.2 Parameters Analyzed

We focused on three parameter groups to analyze:

1. Core eligibility parameters—the basic, widely accepted criteria that determine if a patient should be referred for a presurgical evaluation or not [14]. These parameters were binary and included: Does the patient have focal epilepsy, are they drug resistant, and do they have absolute contraindications for surgery. For the purpose of this study, we utilized the International League Against Epilepsy (ILAE) consensus definition, instructing the models to classify epilepsy as drug‐resistant if there was a failure of adequate trials of two tolerated, appropriately chosen, and used antiepileptic drug schedules (whether as monotherapies or in combination) to achieve sustained seizure freedom [15]. An additional criterion for the purpose of this study was whether the patient had already undergone epilepsy surgery, aligning with the focus of the current study—to identify patients in whom surgery was not utilized or considered. The final parameter in this set was whether the patient is “generally eligible for surgery”, a conclusive binary parameter that is based on the other parameters in this set.
2. Components of the Seizure Freedom Scale (SFS) the SFS is a previously validated composite score developed to predict seizure outcomes after resective epilepsy surgery. It has been shown to correlate with long‐term surgical outcomes in its original cohort and has since undergone external validation, confirming high correlations [16, 17]. The SFS is based on four preoperative factors: (1) Absence of an MRI lesion (restricted to potentially epileptogenic lesions, including but not limited to hippocampal sclerosis, malformations of cortical development, tumors, vascular malformations, and encephalomalacia from infarction, trauma, or infection. Not included are nonspecific MRI findings such as white matter changes or nonspecific overall brain atrophy); (2) High seizure frequency (over 20 seizures per month); (3) Long duration of epilepsy with a cutoff of over 5 years; (4) A history of focal to bilateral tonic–clonic seizures (FBTCS). These components were scored in a uniform direction, with a value of 1 indicating a favorable prognostic feature and 0 an unfavorable feature, in accordance with the original score definition. The model was instructed to extract the four binary SFS parameters and automatically calculate the final composite score (range 0–4, higher indicating more favorable surgical candidacy), matching the manual scoring procedure.

SFS parameters and scores were evaluated only in patients deemed eligible for presurgical evaluation (based on the core eligibility criteria), to reflect a real‐world clinical workflow. Evaluation of the final SFS score required exact agreement with the reference standard, and predictions differing from the true score, regardless of magnitude (e.g., 2 vs. 3 or 2 vs. 4), were considered incorrect. To complement this strict evaluation, performance was also assessed at the level of individual SFS components.

3. Completed Evaluations—parameters related to already‐completed workup evaluations which are usually an integral part of the presurgical process: Was an fMRI completed functional MRI (fMRI) for presurgical functional mapping (e.g., language or motor localization); Is brain MRI (not limited to a specific protocol) recent (< 2y); Was a neuropsychological evaluation completed; Were FDG‐PET or SPECT completed; Was video‐EEG monitoring performed.

4. Prior Surgical Consideration, Patient Stance, History of Surgery—To assess the clinical yield of the screening tool and minimize redundant notifications for patients who are not viable candidates for referral, we analyzed three additional parameters: Has epilepsy surgery been considered previously? (To identify patients previously flagged by clinicians). Has the patient previously refused surgery? (To identify a possible reason for stopping a potential surgical evaluation). Has the patient already undergone epilepsy surgery? (To exclude patients who have already received the intervention).

### 2.3 Manual Review

All cases were manually reviewed by two neurologists, both working and regularly treating patients at the outpatient epilepsy clinical service in Sheba Medical Center. Any inconclusive cases were decided upon after a team discussion involving 3 neurologists (with the institution's head of epilepsy services). Human manual review was deemed the gold standard to which LLM performance was compared.

### 2.4 Large Language Models (LLMs) Used

We selected several widely used state‐of‐the‐art LLMs to allow comparative evaluation. We combined frontier (“Pro”) and efficiency‐oriented (“Flash/mini”) variants from two widely used model families (Gemini and GPT) to balance performance, cost, and practicality. The final models included were from two leading families:

Gemini Models—Gemini 2.5 Pro, Gemini 2.5 Flash, Gemini 2.0 Flash.

GPT Models—GPT‐5, GPT‐5 mini, OpenAI o4‐mini.

All analyses were performed on offline deployments of these models, within Sheba Medical Center's institutional AI infrastructure as part of a hospital‐wide AI innovation initiative. This ensured that patient information remained within the hospital infrastructure and was not transmitted externally.

Default inference settings (e.g., temperature, top_p, n) were chosen to keep a good balance of helpfulness, safety, and creativity.

### 2.5 Prompt Engineering

A structured approach was adopted to minimize variability and improve the quality of the generated responses. The prompts were designed to be unambiguous. A standardized system prompt instructed each model to act as an expert epilepsy neurologist. Zero‐shot prompting was used, where the models were given a single instruction without any examples to avoid pre‐existing bias. Each prompt included explicit instructions detailing the required format for the output.

Prompts used can be seen in the Table S1.

### 2.6 Pilot Study and Prompt Refinement

Prior to conducting an evaluation of the LLM's performance on the validation cohort, a pilot study was conducted to identify potential issues and refine the prompt engineering strategy. Prompts were executed on the pilot cohort of 16 patients. The outputs from each LLM were reviewed and compared with the gold standard data from the manual analysis.

Based on the pilot analysis, several adjustments were made. Prompts were rewritten to be more explicit and unambiguous, and constraints were added to guide the models' outputs more effectively (for instance, more limitations for classifying epilepsy as focal to improve specificity, specifying absolute contraindications for surgery, and clarifying the rules for DRE). Additionally, to prevent inconsistent results and to eliminate LLM hallucinations, each patient's visit summary was processed 3 times independently, and the majority output was determined as the final result. This approach was chosen over reliance on low‐temperature inference, in which LLM results may still vary slightly. Repeated sampling with aggregation helps mitigate stochastic variability or outlier responses.

### 2.7 Evaluation of the LLM'S Performance on the Validation Cohort

Following exclusion of the pilot study cases, 94 cases were included in the validation full‐scale analysis. All of the patient clinical notes, excluding those from the patients included in the pilot phase, were subjected to LLM tagging. The outputs of all six models were systematically compared against the manual tagging to assess performance.

Model performance was evaluated primarily using sensitivity, specificity, positive predictive value (PPV), and negative predictive value (NPV). Confidence intervals (95%) for sensitivity and specificity were calculated using bootstrapping (5,000 resampling iterations). Additional performance metrics, including F1 scores, were calculated and are reported in the Table S1 to allow comparison with prior machine learning studies.

In addition to individual model evaluations, we also performed a consensus analysis: For each case, a majority vote method was applied across the six models. If the majority of models agreed on a classification, that result was adopted. In case of a tie, the result given by the model with the highest F1 score in the primary analysis was used as the tiebreaker.

For instances of missing data, a conservative classification strategy was used. If a clinical note did not contain sufficient information to definitively establish DRE, the patient was classified as not DRE by the human analysis. Similarly, the LLMs were instructed to classify the patient as negative for surgical candidacy.

### 2.8 AI Usage

The authors acknowledge the use of AI‐based writing assistants to improve the language and readability of this manuscript. This assistance was limited to copy‐editing. All scientific concepts, results, and conclusions are the original work of the authors, who assume full responsibility for the accuracy of the manuscript.

## 3 Results

### 3.1 Patient Characteristics

The mean age of the 94 patients (54% female) in the test dataset was 42.3 years (range: 17–90). Table 1 shows demographic and clinical characteristics of the patients. Notably, 29 patients met general eligibility criteria for surgery as assessed by human review, in 13 of whom no prior record of surgical consideration or discussion was evident. This corresponds to a class distribution of approximately 31% eligible vs. 69% non‐eligible patients, indicating moderate class imbalance.

**TABLE 1:** Patient characteristics.

Age (years ± SD)	42.3 (±18.4)
Male (%)	46%
Focal epilepsy (%)	77%
Drug resistant (%)	36%
Absolute contraindications (%)	0%
Generally eligible for surgery (%, *n*)	26%, 29
Already considered for surgery (%, *n*)	15%, 16
Previous epilepsy surgery (%)	6%

Additionally, 16 patients in the cohort had a prior record of surgical evaluation. Of these, 7 patients had ultimately undergone epilepsy surgery, including 6 resective surgeries (e.g., temporal lobectomy, amygdalo‐hippocampectomy, or lesionectomy) and 1 laser interstitial thermal therapy (LITT). At the most recent follow‐up, 3 of these patients were seizure‐free, while 4 had persistent or recurrent seizures. We compared patients deemed eligible for surgical evaluation with and without prior surgical consideration (*n* = 16 vs. *n* = 13). Mean age was slightly higher in patients previously considered (46.8 vs. 37.0 years), while sex distribution was similar (62% vs. 46% male). A structural lesion on MRI was present in 62.5% of patients with prior consideration and 38.5% of those without, with the remainder having no clear lesion. Epilepsy duration exceeded 5 years in nearly all patients in both groups.

### 3.2 Model Performance in Identifying Patients Generally Eligible for Epilepsy Surgery and Core Eligibility Parameters

Table 2 presents the performance of individual models in identifying patients generally eligible for epilepsy surgery, the primary clinically relevant outcome derived from the combination of core eligibility parameters (focal epilepsy, drug resistance, absence of absolute contraindications, and no prior surgery). Overall, models demonstrated high accuracy, with most achieving both high sensitivity and specificity. GPT‐5 mini showed the best overall performance, with sensitivity of 0.95 and specificity of 0.96, along with favorable predictive values (PPV 0.88, NPV 0.99). Other models demonstrated comparable performance.

**TABLE 2:** Performance of individual models and majority vote in identifying patients eligible for epilepsy surgery and core eligibility parameters.

Method	Category	Sensitivity (95% CI)	Specificity (95% CI)	PPV	NPV
Gemini 2.0 Flash	Overall eligibility	0.86 (0.71–1.00)	0.83 (0.74–0.92)	0.61	0.95
Gemini 2.5 Flash	Overall eligibility	0.82 (0.64–0.96)	0.89 (0.82–0.96)	0.69	0.94
Gemini 2.5 Pro	Overall eligibility	0.91 (0.77–1.00)	0.89 (0.81–0.96)	0.71	0.97
OpenAI o4‐mini	Overall eligibility	0.95 (0.85–1.00)	0.92 (0.85–0.97)	0.78	0.99
GPT‐5 mini	Overall eligibility	0.95 (0.84–1.00)	0.96 (0.91–1.00)	0.88	0.99
GPT‐5	Overall eligibility	0.77 (0.57–0.94)	0.97 (0.93–1.00)	0.89	0.93
Majority vote	Has focal epilepsy	0.97 (0.93–1.00)	0.86 (0.70–1.00)	0.96	0.90
Majority vote	Is drug‐resistant	1.00 (1.00–1.00)	0.90 (0.83–0.97)	0.84	1.00
Majority vote	Has absolute contraindications	NA	1.00 (1.00–1.00)	NA	1.00
Majority vote	Had epilepsy surgery	1.00 (1.00–1.00)	1.00 (1.00–1.00)	1.00	1.00
Majority vote	Overall eligibility	1.00 (1.00–1.00)	0.96 (0.91–1.00)	0.88	1.00

*Note:* Performance of individual models and inter‐model consensus (majority vote) in identifying patients eligible for epilepsy surgery and core eligibility parameters. Metrics include sensitivity, specificity, positive predictive value (PPV), and negative predictive value (NPV), with 95% confidence intervals calculated for sensitivity and specificity. Sensitivity and PPV were not estimable for “Has absolute contraindications” as no positive cases were present in the dataset. Cells highlighted in green indicate the highest values among individual models in each column; cells highlighted in red indicate the lowest values among individual models. Majority vote results are not included in this comparison.

At the level of individual core eligibility parameters, models also demonstrated consistently high performance, as detailed in Table S1.

Table 2 also details the consensus results reached by majority vote on each core eligibility parameter. This method achieved high performance in the conclusive decision for overall surgical eligibility, with a sensitivity of 1.00 and specificity of 0.96. PPV and NPV were 0.88 and 1.00, respectively.

### 3.3 Performances in Seizure Freedom Scale Parameter Identification

All models demonstrated high performance in extracting the four parameters of the SFS. Table 3 shows the average performances of each model. The best performing model was GPT‐5 with a sensitivity of 0.91 and a specificity of 0.92. Performance in all components of the SFS by each model is detailed in Table S2 Table 3 also details the consensus results by majority vote on each component of the SFS. Using this method yielded a sensitivity of 0.95 and a specificity of 0.93 in determining the final SFS score.

**TABLE 3:** Performance of individual models and majority vote in seizure freedom scale (SFS) identification.

Method	Category	Sensitivity (95% CI)	Specificity (95% CI)	PPV	NPV
Gemini 2.0 Flash	Overall SFS score	0.73 (0.52–0.90)	0.71 (0.44–0.91)	0.73	0.84
Gemini 2.5 Flash	Overall SFS score	0.86 (0.70–1.00)	0.91 (0.74–1.00)	0.89	0.90
Gemini 2.5 Pro	Overall SFS score	0.86 (0.71–1.00)	0.86 (0.62–1.00)	0.86	0.92
OpenAI o4‐mini	Overall SFS score	0.82 (0.64–0.96)	0.91 (0.73–1.00)	0.89	0.88
GPT‐5 mini	Overall SFS score	0.86 (0.70–1.00)	0.86 (0.65–1.00)	0.86	0.92
GPT‐5	Overall SFS score	0.91 (0.77–1.00)	0.92 (0.74–1.00)	0.91	0.94
Majority vote	MRI normal	1.00 (1.00–1.00)	1.00 (1.00–1.00)	1.00	1.00
Majority vote	FBTCS history	1.00 (1.00–1.00)	1.00 (1.00–1.00)	1.00	1.00
Majority vote	Epilepsy duration > 5y	1.00 (1.00–1.00)	0.95 (0.83–1.00)	0.50	1.00
Majority vote	Seizure frequency > 20/month	1.00 (1.00–1.00)	1.00 (1.00–1.00)	1.00	1.00
Majority vote	Overall SFS score	0.95 (0.85–1.00)	0.93 (0.76–1.00)	0.94	0.98

*Note:* Performance of individual models and inter‐model consensus (majority vote) in identifying Seizure Freedom Scale (SFS) components and overall score. Metrics include sensitivity, specificity, positive predictive value (PPV), and negative predictive value (NPV), with 95% confidence intervals calculated for sensitivity and specificity. Cells highlighted in green indicate the highest values among individual models in each column; cells highlighted in red indicate the lowest values among individual models. Majority vote results are not included in this comparison.

### 3.4 Performance in Recognizing Completed Presurgical Evaluations

All models performed well overall in recognizing which presurgical evaluations had already been completed. Table 4 depicts consensus agreement by majority vote. Performance varied across parameters, with lower sensitivity observed for identifying recent MRI (< 2 years) (sensitivity 0.80, specificity 0.95), while other evaluations, such as video‐EEG monitoring, demonstrated high accuracy (sensitivity 0.88, specificity 0.92). The full data regarding each model's performance on each parameter in this group is displayed in Table S3.

**TABLE 4:** Majority vote results in identifying completed evaluations.

Category	Sensitivity (95% CI)	Specificity (95% CI)	PPV	NPV
Was video‐EEG monitoring performed	0.88 (0.69–1.00)	0.92 (0.71–1.00)	0.93	0.85
Is the brain MRI recent (< 2y)	0.80 (0.33–1.00)	0.95 (0.85–1.00)	0.80	0.95
Were FDG‐PET or SPECT completed	1.00 (1.00–1.00)	0.94 (0.81–1.00)	0.91	1.00
Was a neuropsychological evaluation completed	1.00 (1.00–1.00)	1.00 (1.00–1.00)	1.00	1.00
Was an fMRI completed	1.00 (1.00–1.00)	1.00 (1.00–1.00)	1.00	1.00

*Note:* Consensus results by majority vote method in correctly identifying workup tests completed by patients, which are usually an integral part of the presurgical process.

### 3.5 Performance in Identifying Previous Surgical Consideration, Patient Stance, and Surgical History

The models demonstrated moderate to high abilities in extracting information regarding surgical consideration, patient stance, and whether surgery had already been performed. As detailed in Table 5, the consensus analysis achieved high accuracy for identifying prior surgical consideration (sensitivity 1.00, specificity 1.00), as well as for detecting patient refusal (sensitivity 0.91, specificity 0.90) and history of epilepsy surgery (both sensitivity and specificity 1.00). The full data regarding each model'smodels'’ performance on each parameter in this group is displayed in Table S4.

**TABLE 5:** Accuracy in detecting prior surgical consideration and patient stance.

CAtegory	Sensitivity (95% CI)	Specificity (95% CI)	PPV	NPV
Surgery already considered	1.00 (1.00–1.00)	1.00 (1.00–1.00)	1.00	1.00
Surgery rejected by patient	0.91 (0.78–1.00)	0.90 (0.83–0.96)	0.75	0.97
Surgery already performed	1.00 (1.00–1.00)	1.00 (1.00–1.00)	1.00	1.00

*Note:* Consensus results by majority vote method in correctly identifying whether surgery had already been considered, whether the patient expressed a negative stance towards surgery, and whether surgery had already been undergone.

## 4 Discussion

This study's findings show that LLMs can identify patients eligible for epilepsy surgery evaluation, and further stratify them prognostically by extracting relevant information from unstructured clinical notes. The models achieved high sensitivity and specificity in identifying surgical eligibility (up to 0.95 and 0.96, respectively), with strong positive and negative predictive values (up to 0.88 and 0.99, respectively), minimizing missed candidates while maintaining reasonable precision. An inter‐model voting demonstrated even higher results, as depicted in Table 2.

Notably, 45% of eligible patients in this cohort had not been previously considered for surgery, a finding consistent with the well‐described underutilization and delays in epilepsy surgery.

Together, these findings suggest that automated analysis of clinical notes can help address gaps in the recognition of surgical candidates. Systematic identification of patients through decision‐support tools can complement, rather than replace, clinical judgment and lead to more consistent referral practices. In practical terms, these tools could integrate into routine clinical workflows to prompt reconsideration of surgical referral when appropriate, or be used for periodic retrospective auditing of EHRs to identify overlooked cases. In this cohort, this approach could have uncovered 13 unrecognized surgical candidates, a 45% increase in eligible patients. Notably, these patients did not differ substantially in clinical characteristics from those already recognized, although structural lesions were more frequent among previously considered patients; many overlooked individuals also had identifiable MRI abnormalities. This suggests that under‐referral reflects inconsistent recognition rather than different patient profiles.

Beyond binary identification of surgical eligibility, extracting prognostic parameters can allow for stratifying candidates to prioritize referrals. This could also help reduce the risk of over‐referral, which may subject patients to unnecessary evaluations. For this purpose, the SFS, a validated prognostic tool for predicting which patients would ultimately undergo surgery and have favorable results, was applied and was correctly scored by the models (Table 3). Furthermore, identification of previously completed presurgical investigations and prior surgical consideration may further improve the efficiency and yield of screening efforts (Table 4 and Table 5).

These potential applications may provide a pathway for clinically meaningful practice changes. However, prospective studies are needed to evaluate the impact on referral amounts, referral appropriateness, and patient outcomes.

### 4.1 Positioning in the Context of Prior Work: From Feature Engineering to Universal Contextual Understanding

The present LLM‐based approach is notable in the context of prior avenues of automated screening for surgical candidates. Several important studies, including those by Wissel et al., have demonstrated the feasibility of such approaches, progressing from designing rule‐based and n‐gram models to ultimately integrating them into electronic health records (EHRs) as automated alerts, showing the potential to increase surgical referral rates [9, 18, 19, 20]. More recently, Tan et al. integrated LLMs into this workflow, yet retained a traditional Random Forest model for the core task of candidate identification, using LLMs only for summarizing the clinical notes post‐identification [21]. These approaches remain tied to supervised learning techniques, require extensive feature engineering and site‐specific training, and are inherently language‐specific.

The LLM performances in this study are consistent with prior work demonstrating the capability of LLMs to extract structured clinical information from unstructured text, while extending this to a clinically relevant task of surgical candidacy assessment. Recent advances in LLM development have also led to the emergence of domain‐specific models trained or fine‐tuned for medical reasoning and diagnosis, such as Med‐PaLM 2 and MedFound, which demonstrate strong performance on medical question‐answering benchmarks and clinical diagnostic tasks [12, 22]. These models are designed to incorporate domain‐specific knowledge and emulate clinical reasoning processes.

In contrast, our study assesses whether off‐the‐shelf LLMs can achieve these tasks directly from raw, unstructured narratives using zero‐shot prompting, without the need for structured inputs, feature engineering, or retraining, and in languages other than English. This approach represents a shift towards using semantic understanding rather than predefined text patterns, with significant potential advantages in portability and ease of implementation.

In parallel, general‐purpose LLMs continue to play a central role in clinical research due to their strong zero‐shot capabilities and ability to process heterogeneous clinical narratives without task‐specific training, often achieving performance comparable to domain‐specific systems [23].

In this context, our findings suggest that widely available general‐purpose models can support clinically relevant screening tasks directly from raw, non‐English clinical text, offering a complementary and potentially more accessible approach. This paradigm may be particularly advantageous in settings where the development or deployment of domain‐specific models is impractical and supports the feasibility of large‐scale screening across diverse healthcare systems.

## 5 Limitations

This study has several limitations. The models were not tested prospectively in a clinical setting, and their retrospectively demonstrated accuracies are not yet shown to have real‐world clinical impact. Future research should evaluate the impact of prospective clinical implementation on surgical referrals and patient outcomes.

Furthermore, the study sample was relatively small and derived from a single tertiary academic epilepsy center. Findings may not fully generalize to non‐specialist or community settings, where clinical documentation and patient populations may differ; therefore, further studies should strive for multi‐center and potentially international validation using a larger dataset. Additionally, the relatively small number of eligible patients introduces class imbalance, which may influence performance metrics and should be considered when interpreting the results.

Other limitations include: None of the patients in the cohort had absolute contraindications to surgery, limiting evaluation of model performance for this parameter; MRI evaluations were not assessed for adherence to the ILAE‐recommended HARNESS protocol; individual outputs from repeated model runs were not retained, precluding analysis of intra‐model agreement (e.g., 3:0 vs. 2:1 splits) and its relationship to prediction accuracy; LLM development is dynamic, and current available models may become obsolete in the future, although model capabilities are expected to improve over time.

## 6 Conclusions

This study demonstrates that LLMs can be used to identify and stratify patients who may be eligible for epilepsy surgery evaluation, offering a potential scalable approach to address the under‐utilization of epilepsy surgery. Practical applications could include integration as decision‐support tools to flag surgical candidates during routine care, or automated auditing of EHRs to detect patients who may have been previously overlooked. The goal of such systems is not to replace clinician judgment, but to reduce missed referral opportunities by systematically identifying patients who meet established surgical criteria.

The clinical applicability is supported by consistently high model performance, including high sensitivity and accuracy across eligibility parameters, achieved using widely available off‐the‐shelf models without task‐specific training. This zero‐shot approach reduces the barrier to implementation and suggests feasibility across diverse clinical settings, including non‐English speaking environments.

## Author Contributions

Dr. Uriel Fennig (Corresponding Author): Lead conceptualization of the study; assignment of research roles; writing the majority of the original draft; overall supervision. Dr. Nadav Amir: Technical implementation of the Large Language Models; programming and software handling; data analysis. Dr. Maya Schiller: Execution of human validation processes; significant contribution to drafting and editing the manuscript. Dr. Roni Loebenstein: Assistance in study conceptualization and design; expert epileptology consultation; review and editing of the manuscript. Dr. Johnatan Nissan: Statistical analysis and consultation; contribution to general study design and methodology. Dr. Marina Boxer: Participation in original conceptualization; expert epileptology consultation; review and editing of results and the manuscript. Dr. Shany Guly Gofrit: Statistical analysis; consultation regarding result significance; manuscript review and editing. Dr. Gal Goshen: Coordination of the framework for clinical note extraction from EHRs; creation of the coding workstation; management of LLM access and acquisition; study design and concept refinement. Prof. Sándor Beniczky: Extensive review and re‐review of manuscript drafts; major contribution in study design and analysis. Prof. Nicola Maggio: Conceptualization and study design; coordination of the research workflow; supervision of human validation.

## Funding

The authors have nothing to report.

## Ethics Statement

The study protocol was reviewed and approved by the institutional ethics committee (Sheba Medical Center Helsinki Committee No. 1277–24), with a waiver of individual informed consent granted owing to the retrospective design and locally processed nature of the analysis.

## Conflicts of Interest

The authors declare no conflicts of interest.

## Supporting information


**Table S1:** Core Eligibility Criteria Assessment by Model.
**Table S2:** SFS Component Assessment by Model.
**Table S3:** Assessment of Presurgical Evaluations Completed by Model.
**Table S4:** Assessment of Whether Surgical Option Was Already Discussed by Model.

## Data Availability

The data that support the findings of this study are available on request from the corresponding author. The data are not publicly available due to privacy or ethical restrictions.
