# Supplementary material for: Screening Routine Clinical Notes for Epilepsy Surgery Candidates Using Large Language Models
Source: Ann Clin Transl Neurol. 2026 May 5:10.1002/acn3.70427. Online ahead of print. doi: 10.1002/acn3.70427 (PMC13394138; doi:10.1002/acn3.70427)
Supplement: Supplementary file 1 — Table S1: Core Eligibility Criteria Assessment by Model. Table S2: SFS Component Assessment by Model. Table S3: Assessment of Presurgical Evaluations Completed by Model. Table S4: Assessment of Whether Surgical Option Was Already Discussed by Model. [file ACN3-9999-0-s001.docx]

Supplementary Material

Prompts used

Below are the exact instructional prompts provided to the large language model

**Main System Prompt (General Eligibility and Presurgical Workup Variables)**

Introduction

=============

Today is {current_date}.

You are an expert in epilepsy.

You will be given patient visit summaries to analyze and determine the patient's suitability for epilepsy surgery.

For each patient you will get concatenated visit summaries in chronological order (older to newer visits).

Before reading these summaries you will be given some information about which patients are eligible for surgery

and why.

Drug Resistance Epilepsy - DRE

==============================

Epilepsy is considered drug resistant if there was a failure of adequate trials of two tolerated, appropriately chosen and used antiepileptic drug schedules (whether as mono therapies or in combination) to achieve sustained seizure freedom (ILAE Kwan 2010)

# Take into account whether maximal possible dosages of the antiepileptic drugs were used - if submaximal dosages were used then the patient is not drug resistant (e.g., Keppra 1000mg per day is not considered an adequate drug trial).

# Consider drug resistance only if the patient has failed at least 2 drugs. Failure of one drug, even at maximal dosage, is not considered drug resistance.

# If the patient was drug resistant before and the undergone epilepsy surgery he is still considered drug resistant

# Seizure freedom should be attributed only if patient is seizure free for at least 12 months, and 3 times the length of the pre-intervention period (i.e., before the last drug was started).

Absolute contraindications for surgery

=======================================

Timing of referral to evaluate for epilepsy Surgery: expert consensus recommendations from the surgical therapies commission of the International League Against Epilepsy (ILAE Jehi 2022):

- Referral for surgery should not be offered to patients with active substance abuse who are non-cooperative with management.

Pre-surgical workup

====================

Sometimes pre-surgical workup should be completed (for possibly eligible patients only):

- Better clinical history

- MRI or repeating MRI

- Video EEG

- Neuropyschological evaluation

- fMRI

Sometimes further advanced testing is needed to evaluate eligibility for surgery, possible tests are:

- Interictal FDG-PET

- Ictal SPECT

- Postprocessing MRI

- Intracranial stereo-EEG

- Intracranial surface EEG (Grids/Strips)

- EEG source localization (HD-EEG)

- MEG

- Wada test

There are multiple reasons further advanced testing might be needed (in addition to the basic pre-surgical workup):

- Optionally recommended for:

- Hippocampal Sclerosis

- Discrete tumors

- Focal Cortical dysplasia type II

- AVM

- Stroke

- Sturge-Weber syndrome

- Highly recommended for:

- Focal Cortical Dysplasia type I

- Tuberous sclerosis

- Multilesional cases

- Discordant findings between video-EEG and MRI

- Close spatial relationship between hypothesized epileptogenic zone and eloquent cortex

- Non-lesional cases (imaging negative)

Focal Epilepsy

===============

Here are some thumb rules for tagging focal epilepsy:

# Focal epilepsy should be tagged only if there is proof for it - focal activity in the eeg or semiology that strongly suggests it. Otherwise consider the epilepsy to be non focal.

# Don't base your answer on MRI findings alone! Even if they strongly suggest focal epilepsy.

# If the patient has experienced only a single event, that has a differential diagnosis in which a seizure is not clearly the leading cause (e.g. the event might have been a TIA) do not consider this epilepsy.

# If EEG recordings have shown general discharges, or if an epilepsy syndrome was diagnosed which is characterized by generalized epilepsy, consider as not focal even if semiology might suggest otherwise.

# Multifocal epilepsy should be tagged as focal epilepsy.

# If the patient possibly doesn't have epilepsy at all don't consider as focal epilepsy.

Response format

===============

Now you will start processing patient visit summaries and determine the patient's suitability for epilepsy surgery based on multiple scores as will be explained soon.

The scoring should be based on given patient visit summaries - each message will contain the concatenated patient visit summaries from different visits to the epilepsy clinic in chronological order (older to newer visits).

Provide your response in the following JSON format:

{

"patient_id": <patient id>,

"gender": <int - Male=1 and Female=0, if unknown return null>,

"age": <int - How old is the patient - don't estimate the age today! simply return the mentioned age and if it is not available return null>,

"number_of_visits": <int - the amount of visits to the epilepsy clinic>,

"is_focal_epilepsy": <boolean - does the patient have focal epilepsy? take into account the rules mentioned previously in the "Focal Epilepsy" section - classify as focal only if there is strong evidence towards it>,

"is_focal_epilepsy_description": <text - explanation for the is_focal_epilepsy score, reference the "Focal Epilepsy" section rules>,

"is_drug_resistant": <boolean - whether the epilepsy is drug resistant as defined earlier, take into consideration newer visits before older visits - if there were events in newer visits then the patient is not drug resistant>,

"is_drug_resistant_description": <string - the explanation for the is_drug_resistant score>,

"has_absolute_contraindications": <boolean - is there progressive disease, severe cognitive impairment (mild cognitive impairment doesn't count), or active substance abuse (if the patient stopped using it is not a contraindication anymore)>,

"has_absolute_contraindications_description": <text - what is the absolute contraindication for surgery>,

"is_after_epilepsy_surgery": <boolean - whether the patient has already undergone epilepsy surgery - don't take into account Neurostimulation Device Implantations like VNS or drug pumps insertion - those are still considered False!>,

"surgery_already_considered": <boolean - whether surgery was already considered and discussed with the patient - don't take into account Neurostimulation Device Implantations like VNS or drug pumps insertion>,

"surgery_rejected_by_patient": <boolean - if surgery_already_considered was true, whether the patient rejected the surgical option>

"after_what_epilepsy_surgery": <text - if is_after_epilepsy_surgery is true then what surgery is the patient after?>,

"is_generally_eligible_for_surgery": <boolean - true if is_drug_resistant is true and is_focal_epilepsy is true; false if generalized or multifocal epilepsy, not drug-resistant, has_absolute_contraindications is true or is_after_epilepsy_surgery is true>,

"mri_lesion_type": <string - Specify lesion type if present (Hippocampal Sclerosis, LEAT, FCD, Cavernoma, MRI-negative, Suspected-but-inconclusive-changes, Other)>,

"mri_lesion_type_details": <string - Elaborate on lesion type if present (a description lesion type and localisation>,

"was_video_eeg_completed": <boolean - Has video EEG monitoring been performed? use false if performed but did not capture electroclinical events>,

"is_mri_recent": <boolean - Has MRI or repeat MRI been done in the last 2 years (be strict! 2 years and one month is not considered recent)>,

"last_mri_estimated_date" : <string - When do you estimate the patient had his last MRI?>,

"was_pet_spect_completed": <boolean - Whether PET/SPECT been completed if needed>,

"was_neuropsychological_evaluation_completed": <boolean - Whether a neuropsychological evaluation been done>,

"was_fmri_completed": <boolean - Whether functional MRI (fmri) been conducted>,

"surgical_readiness": <float - How complete the presurgical workup is: the ratio of how many of the last 5 variables were true (out of potential 5) - don't show the ratio, please give a simple float>

}

Please be strict! Return these fields only!

**SFS-Specific Prompt**

Introduction

=============

Today is {current_date}.

You are an expert in epilepsy.

You will be given patient visit summaries to analyze and determine the patient's suitability for epilepsy surgery.

For each patient you will get concatenated visit summaries in chronological order (older to newer visits).

Before reading these summaries you will be given some information about which patients are eligible for surgery and why.

Response format

===============

Now you will start processing patient visit summaries and determine the patient's suitability for epilepsy surgery based on multiple scores as will be explained soon.

The scoring should be based on given patient visit summaries - each message will contain the concatenated patient visit summaries from different visits to the epilepsy clinic in chronological order (older to newer visits).

Provide your response in the following JSON format:

{

"patient_id": <patient id>,

"is_mri_normal": <int - Is the mri normal - 0 yes, 1 no, if unclear or if unsure return 0>,

"gtcs_history": <int - Is there a history of GTCS ever (including focal-to-bilateral tonic–clonic seizures) - 0 yes, 1 no, if unclear or if unsure return 1>,

"epilepsy_duration_long": <int - Is the duration of epilepsy over 5 years - 0 yes, 1 no, if unclear or if unsure return 0>,

"high_frequency_seizures": <int - Are there more than 20 seizures per month according to last visit only - 0 yes or probably, 1 no or probably not, if unclear or if unsure return 1>,

"sfs_score": <int - The sum of is_mri_normal+gtcs_history+epilepsy_duration_long+high_frequency_seizures>

}

Please be strict! Return these fields only!

Supplementary Material

Supplementary Table 1 - Core Eligibility Criteria Assessment by Model

| **model** | **category** | **agreement(%)** | **sensitivity (95% CI)** | **specificity (95% CI)** | **ppv** | **npv** | **F1 (95% CI)** | **kappa (95% CI)** |
| --- | --- | --- | --- | --- | --- | --- | --- | --- |
| Gemini 2.0 Flash | Has focal epilepsy | 86.00 | 0.94(0.89-0.99) | 0.59(0.38-0.80) | 0.88 | 0.76 | 0.91(0.86-0.96) | 0.58(0.38-0.78) |
| Gemini 2.0 Flash | Is drug resistant | 86.00 | 1.00(1.00-1.00) | 0.79(0.68-0.89) | 0.71 | 1.00 | 0.83(0.73-0.91) | 0.72(0.58-0.86) |
| Gemini 2.0 Flash | Has absolute contraindications | 96.00 |  | 0.97(0.93-1.00) |  | 1.00 |  |  |
| Gemini 2.0 Flash | Had epilepsy surgery | 100.00 | 1.00(1.00-1.00) | 1.00(1.00-1.00) | 1.00 | 1.00 | 1.00(1.00-1.00) | 1(1-1) |
| Gemini 2.0 Flash | Is generally eligible for surgery | 84.00 | 0.86(0.71-1.00) | 0.83(0.74-0.92) | 0.61 | 0.95 | 0.72(0.55-0.84) | 0.61(0.44-0.78) |
| Gemini 2.5 Flash | Has focal epilepsy | 92.00 | 0.97(0.93-1.00) | 0.77(0.58-0.94) | 0.93 | 0.89 | 0.95(0.91-0.98) | 0.78(0.63-0.94) |
| Gemini 2.5 Flash | Is drug resistant | 85.00 | 0.88(0.75-0.97) | 0.84(0.74-0.93) | 0.74 | 0.93 | 0.80(0.68-0.90) | 0.68(0.53-0.83) |
| Gemini 2.5 Flash | Has absolute contraindications | 100.00 |  | 1.00(1.00-1.00) |  | 1.00 |  | 1(1-1) |
| Gemini 2.5 Flash | Had epilepsy surgery | 100.00 | 1.00(1.00-1.00) | 1.00(1.00-1.00) | 1.00 | 1.00 | 1.00(1.00-1.00) | 1(1-1) |
| Gemini 2.5 Flash | Is generally eligible for surgery | 87.00 | 0.82(0.64-0.96) | 0.89(0.82-0.96) | 0.69 | 0.94 | 0.75(0.59-0.87) | 0.67(0.49-0.84) |
| Gemini 2.5 Pro | Has focal epilepsy | 92.00 | 0.97(0.93-1.00) | 0.77(0.58-0.94) | 0.93 | 0.89 | 0.95(0.91-0.98) | 0.78(0.63-0.94) |
| Gemini 2.5 Pro | Is drug resistant | 87.00 | 0.97(0.90-1.00) | 0.82(0.72-0.91) | 0.74 | 0.98 | 0.84(0.74-0.92) | 0.74(0.6-0.87) |
| Gemini 2.5 Pro | Has absolute contraindications | 98.00 |  | 0.99(0.97-1.00) |  | 1.00 |  |  |
| Gemini 2.5 Pro | Had epilepsy surgery | 100.00 | 1.00(1.00-1.00) | 1.00(1.00-1.00) | 1.00 | 1.00 | 1.00(1.00-1.00) | 1(1-1) |
| Gemini 2.5 Pro | Is generally eligible for surgery | 89.00 | 0.91(0.77-1.00) | 0.89(0.81-0.96) | 0.71 | 0.97 | 0.80(0.65-0.90) | 0.73(0.57-0.88) |
| GPT O4 Mini | Has focal epilepsy | 95.00 | 0.97(0.93-1.00) | 0.91(0.77-1.00) | 0.97 | 0.91 | 0.97(0.94-0.99) | 0.88(0.77-0.99) |
| GPT O4 Mini | Is drug resistant | 92.00 | 0.97(0.90-1.00) | 0.90(0.82-0.97) | 0.84 | 0.98 | 0.90(0.81-0.97) | 0.84(0.73-0.95) |
| GPT O4 Mini | Has absolute contraindications | 100.00 |  | 1.00(1.00-1.00) |  | 1.00 |  | 1(1-1) |
| GPT O4 Mini | Had epilepsy surgery | 100.00 | 1.00(1.00-1.00) | 1.00(1.00-1.00) | 1.00 | 1.00 | 1.00(1.00-1.00) | 1(1-1) |
| GPT O4 Mini | Is generally eligible for surgery | 92.00 | 0.95(0.85-1.00) | 0.92(0.85-0.97) | 0.78 | 0.99 | 0.86(0.73-0.95) | 0.81(0.67-0.94) |
| GPT 5 Mini | Has focal epilepsy | 94.00 | 0.97(0.93-1.00) | 0.86(0.70-1.00) | 0.96 | 0.90 | 0.97(0.93-0.99) | 0.85(0.72-0.98) |
| GPT 5 Mini | Is drug resistant | 93.00 | 1.00(1.00-1.00) | 0.90(0.83-0.97) | 0.84 | 1.00 | 0.91(0.84-0.97) | 0.86(0.76-0.97) |
| GPT 5 Mini | Has absolute contraindications | 98.00 |  | 0.99(0.97-1.00) |  | 1.00 |  |  |
| GPT 5 Mini | Had epilepsy surgery | 100.00 | 1.00(1.00-1.00) | 1.00(1.00-1.00) | 1.00 | 1.00 | 1.00(1.00-1.00) | 1(1-1) |
| GPT 5 Mini | Is generally eligible for surgery | 95.00 | 0.95(0.84-1.00) | 0.96(0.91-1.00) | 0.88 | 0.99 | 0.91(0.81-0.98) | 0.88(0.77-0.99) |
| GPT 5 | Has focal epilepsy | 92.00 | 0.93(0.87-0.99) | 0.91(0.78-1.00) | 0.97 | 0.80 | 0.95(0.91-0.98) | 0.8(0.66-0.94) |
| GPT 5 | Is drug resistant | 90.00 | 0.88(0.76-0.97) | 0.92(0.84-0.98) | 0.85 | 0.93 | 0.86(0.76-0.94) | 0.79(0.66-0.92) |
| GPT 5 | Has absolute contraindications | 100.00 |  | 1.00(1.00-1.00) |  | 1.00 |  | 1(1-1) |
| GPT 5 | Had epilepsy surgery | 100.00 | 1.00(1.00-1.00) | 1.00(1.00-1.00) | 1.00 | 1.00 | 1.00(1.00-1.00) | 1(1-1) |
| GPT 5 | Is generally eligible for surgery | 92.00 | 0.77(0.57-0.94) | 0.97(0.93-1.00) | 0.89 | 0.93 | 0.83(0.68-0.94) | 0.78(0.63-0.94) |
| **Majority Vote** | Has focal epilepsy | 94.00 | 0.97(0.93-1.00) | 0.86(0.70-1.00) | 0.96 | 0.90 | 0.97(0.93-0.99) | 0.85(0.72-0.98) |
| **Majority Vote** | Is drug resistant | 93.00 | 1.00(1.00-1.00) | 0.90(0.83-0.97) | 0.84 | 1.00 | 0.91(0.83-0.97) | 0.86(0.76-0.97) |
| **Majority Vote** | Has absolute contraindications | 100.00 |  | 1.00(1.00-1.00) |  | 1.00 |  | 1(1-1) |
| **Majority Vote** | Had epilepsy surgery | 100.00 | 1.00(1.00-1.00) | 1.00(1.00-1.00) | 1.00 | 1.00 | 1.00(1.00-1.00) | 1(1-1) |
| **Majority Vote** | Is generally eligible for surgery | 96.00 | 1.00(1.00-1.00) | 0.96(0.91-1.00) | 0.88 | 1.00 | 0.94(0.85-1.00) | 0.92(0.82-1.01) |

Supplementary Table 2 - SFS Component Assessment by Model

| **model** | **category** | **agreement (%)** | **sensitivity (95% CI)** | **specificity (95% CI)** | **ppv** | **npv** | **F1 (95% CI)** | **kappa (95% CI)** |
| --- | --- | --- | --- | --- | --- | --- | --- | --- |
| Gemini 2.0 Flash | is_mri_normal | 100.00 | 1.00(1.00-1.00) | 1.00(1.00-1.00) | 1.00 | 1.00 | 1.00(1.00-1.00) | 1(1-1) |
| Gemini 2.0 Flash | gtcs_history | 81.00 |  | 1.00(1.00-1.00) |  | 0.82 |  |  |
| Gemini 2.0 Flash | epilepsy_duration_long | 95.00 |  | 1.00(1.00-1.00) |  | 0.95 |  |  |
| Gemini 2.0 Flash | high_frequency_seizures | 100.00 | 1.00(1.00-1.00) | 1.00(1.00-1.00) | 1.00 | 1.00 | 1.00(1.00-1.00) | 1(1-1) |
| Gemini 2.0 Flash | sfs_score | 72.00 | 0.73(0.52-0.90) | 0.71(0.44-0.91) | 0.73 | 0.84 | **0.68(0.45-0.89)** | 0.81(0.57-1.06) |
| Gemini 2.5 Flash | is_mri_normal | 95.00 | 1.00(1.00-1.00) | 0.83(0.50-1.00) | 0.94 | 1.00 | 0.97(0.89-1.00) | 0.88(0.65-1.11) |
| Gemini 2.5 Flash | gtcs_history | 95.00 | 0.75(0.00-1.00) | 1.00(1.00-1.00) | 1.00 | 0.95 | 0.86(0.50-1.00) | 0.83(0.51-1.15) |
| Gemini 2.5 Flash | epilepsy_duration_long | 100.00 | 1.00(1.00-1.00) | 1.00(1.00-1.00) | 1.00 | 1.00 | 1.00(1.00-1.00) | 1(1-1) |
| Gemini 2.5 Flash | high_frequency_seizures | 95.00 | 0.95(0.82-1.00) | 1.00(1.00-1.00) | 1.00 | 0.75 | 0.97(0.90-1.00) | 0.83(0.51-1.15) |
| Gemini 2.5 Flash | sfs_score | 86.00 | 0.86(0.70-1.00) | 0.91(0.74-1.00) | 0.89 | 0.90 | **0.86(0.70-1.00)** | 0.91(0.79-1.04) |
| Gemini 2.5 Pro | is_mri_normal | 100.00 | 1.00(1.00-1.00) | 1.00(1.00-1.00) | 1.00 | 1.00 | 1.00(1.00-1.00) | 1(1-1) |
| Gemini 2.5 Pro | gtcs_history | 95.00 | 0.75(0.00-1.00) | 1.00(1.00-1.00) | 1.00 | 0.95 | 0.86(0.50-1.00) | 0.83(0.51-1.15) |
| Gemini 2.5 Pro | epilepsy_duration_long | 100.00 | 1.00(1.00-1.00) | 1.00(1.00-1.00) | 1.00 | 1.00 | 1.00(1.00-1.00) | 1(1-1) |
| Gemini 2.5 Pro | high_frequency_seizures | 95.00 | 0.95(0.83-1.00) | 1.00(1.00-1.00) | 1.00 | 0.75 | 0.97(0.91-1.00) | 0.83(0.51-1.15) |
| Gemini 2.5 Pro | sfs_score | 86.00 | 0.86(0.71-1.00) | 0.86(0.62-1.00) | 0.86 | 0.92 | **0.86(0.69-1.00)** | 0.91(0.77-1.05) |
| OpenAI o4-mini | is_mri_normal | 86.00 | 0.94(0.79-1.00) | 0.67(0.25-1.00) | 0.88 | 0.80 | 0.91(0.78-1.00) | 0.64(0.27-1.01) |
| OpenAI o4-mini | gtcs_history | 100.00 | 1.00(1.00-1.00) | 1.00(1.00-1.00) | 1.00 | 1.00 | 1.00(1.00-1.00) | 1(1-1) |
| OpenAI o4-mini | epilepsy_duration_long | 90.00 | 1.00(1.00-1.00) | 0.90(0.76-1.00) | 0.33 | 1.00 | 0.50(0.29-1.00) | 0.46(-0.13-1.06) |
| OpenAI o4-mini | high_frequency_seizures | 100.00 | 1.00(1.00-1.00) | 1.00(1.00-1.00) | 1.00 | 1.00 | 1.00(1.00-1.00) | 1(1-1) |
| OpenAI o4-mini | sfs_score | 81.00 | 0.82(0.64-0.96) | 0.91(0.73-1.00) | 0.89 | 0.88 | **0.84(0.67-0.97)** | 0.89(0.77-1.02) |
| GPT-5 mini | is_mri_normal | 95.00 | 1.00(1.00-1.00) | 0.83(0.43-1.00) | 0.94 | 1.00 | 0.97(0.89-1.00) | 0.88(0.65-1.11) |
| GPT-5 mini | gtcs_history | 100.00 | 1.00(1.00-1.00) | 1.00(1.00-1.00) | 1.00 | 1.00 | 1.00(1.00-1.00) | 1(1-1) |
| GPT-5 mini | epilepsy_duration_long | 95.00 | 1.00(1.00-1.00) | 0.95(0.84-1.00) | 0.50 | 1.00 | 0.67(0.40-1.00) | 0.65(0.01-1.28) |
| GPT-5 mini | high_frequency_seizures | 95.00 | 1.00(1.00-1.00) | 0.67(0.00-1.00) | 0.95 | 1.00 | 0.97(0.91-1.00) | 0.78(0.36-1.19) |
| GPT-5 mini | sfs_score | 86.00 | 0.86(0.70-1.00) | 0.86(0.65-1.00) | 0.86 | 0.92 | **0.86(0.68-1.00)** | 0.91(0.77-1.04) |
| GPT-5 | is_mri_normal | 95.00 | 0.94(0.79-1.00) | 1.00(1.00-1.00) | 1.00 | 0.86 | 0.97(0.89-1.00) | 0.89(0.68-1.1) |
| GPT-5 | gtcs_history | 100.00 | 1.00(1.00-1.00) | 1.00(1.00-1.00) | 1.00 | 1.00 | 1.00(1.00-1.00) | 1(1-1) |
| GPT-5 | epilepsy_duration_long | 95.00 | 1.00(1.00-1.00) | 0.95(0.84-1.00) | 0.50 | 1.00 | 0.67(0.40-1.00) | 0.65(0.01-1.28) |
| GPT-5 | high_frequency_seizures | 100.00 | 1.00(1.00-1.00) | 1.00(1.00-1.00) | 1.00 | 1.00 | 1.00(1.00-1.00) | 1(1-1) |
| GPT-5 | sfs_score | 90.00 | 0.91(0.77-1.00) | 0.92(0.74-1.00) | 0.91 | 0.94 | **0.91(0.77-1.00)** | 0.94(0.84-1.04) |
| Majority Vote | is_mri_normal | 100.00 | 1.00(1.00-1.00) | 1.00(1.00-1.00) | 1.00 | 1.00 | 1.00(1.00-1.00) | 1(1-1) |
| Majority Vote | gtcs_history | 100.00 | 1.00(1.00-1.00) | 1.00(1.00-1.00) | 1.00 | 1.00 | 1.00(1.00-1.00) | 1(1-1) |
| Majority Vote | epilepsy_duration_long | 95.00 | 1.00(1.00-1.00) | 0.95(0.83-1.00) | 0.50 | 1.00 | 0.67(0.40-1.00) | 0.65(0.01-1.28) |
| Majority Vote | high_frequency_seizures | 100.00 | 1.00(1.00-1.00) | 1.00(1.00-1.00) | 1.00 | 1.00 | 1.00(1.00-1.00) | 1(1-1) |
| Majority Vote | sfs_score | 95.00 | 0.95(0.85-1.00) | 0.93(0.76-1.00) | 0.94 | 0.98 | **0.95(0.82-1.00)** | 0.97(0.9-1.04) |

Supplementary Table 3 - Assessment of Presurgical Evaluations Completed by Model

| **Model** | **Category** | **Agreement(%)** | **Sensitivity (95% CI)** | **Specificity (95% CI)** | **PPV** | **NPV** | **F1 (95% CI)** | **Kappa (95% CI)** |
| --- | --- | --- | --- | --- | --- | --- | --- | --- |
| Gemini 2.0 Flash | was video eeg completed | 96.00 | 0.94(0.79-1.00) | 1.00(1.00-1.00) | 1.00 | 0.92 | 0.97(0.89-1.00) | 0.93(0.79-1.07) |
| Gemini 2.0 Flash | is mri recent | 81.00 | 0.80(0.33-1.00) | 0.82(0.64-0.96) | 0.50 | 0.95 | 0.62(0.25-0.89) | 0.5(0.14-0.87) |
| Gemini 2.0 Flash | was pet spect completed | 92.00 | 1.00(1.00-1.00) | 0.89(0.72-1.00) | 0.83 | 1.00 | 0.91(0.74-1.00) | 0.85(0.65-1.05) |
| Gemini 2.0 Flash | was neuropsychological evaluation completed | 96.00 | 1.00(1.00-1.00) | 0.96(0.87-1.00) | 0.67 | 1.00 | 0.80(0.40-1.00) | 0.78(0.37-1.19) |
| Gemini 2.0 Flash | was fmri completed | 100.00 | 1.00(1.00-1.00) | 1.00(1.00-1.00) | 1.00 | 1.00 | 1.00(1.00-1.00) | 1(1-1) |
| Gemini 2.5 Flash | was video eeg completed | 85.00 | 0.88(0.69-1.00) | 0.83(0.60-1.00) | 0.88 | 0.83 | 0.88(0.73-0.98) | 0.71(0.44-0.97) |
| Gemini 2.5 Flash | is mri recent | 92.00 | 0.80(0.33-1.00) | 0.95(0.85-1.00) | 0.80 | 0.95 | 0.80(0.40-1.00) | 0.75(0.43-1.08) |
| Gemini 2.5 Flash | was pet spect completed | 96.00 | 1.00(1.00-1.00) | 0.94(0.82-1.00) | 0.91 | 1.00 | 0.95(0.82-1.00) | 0.92(0.78-1.07) |
| Gemini 2.5 Flash | was neuropsychological evaluation completed | 96.00 | 1.00(1.00-1.00) | 0.96(0.87-1.00) | 0.67 | 1.00 | 0.80(0.50-1.00) | 0.78(0.37-1.19) |
| Gemini 2.5 Flash | was fmri completed | 100.00 | 1.00(1.00-1.00) | 1.00(1.00-1.00) | 1.00 | 1.00 | 1.00(1.00-1.00) | 1(1-1) |
| Gemini 2.5 Pro | was video eeg completed | 85.00 | 0.88(0.69-1.00) | 0.83(0.58-1.00 | 0.88 | 0.83 | 0.88(0.73-0.97) | 0.71(0.44-0.97) |
| Gemini 2.5 Pro | is mri recent | 92.00 | 0.80(0.33-1.00) | 0.95(0.84-1.00 | 0.80 | 0.95 | 0.80(0.40-1.00) | 0.75(0.43-1.08) |
| Gemini 2.5 Pro | was pet spect completed | 96.00 | 1.00(1.00-1.00) | 0.94(0.81-1.00 | 0.91 | 1.00 | 0.95(0.82-1.00) | 0.92(0.78-1.07) |
| Gemini 2.5 Pro | was neuropsychological evaluation completed | 100.00 | 1.00(1.00-1.00) | 1.00(1.00-1.00 | 1.00 | 1.00 | 1.00(1.00-1.00) | 1(1-1) |
| Gemini 2.5 Pro | was fmri completed | 100.00 | 1.00(1.00-1.00) | 1.00(1.00-1.00 | 1.00 | 1.00 | 1.00(1.00-1.00) | 1(1-1) |
| OpenAI o4-mini | was video eeg completed | 96.00 | 0.94(0.80-1.00) | 1.00(1.00-1.00 | 1.00 | 0.92 | 0.97(0.88-1.00) | 0.93(0.79-1.07) |
| OpenAI o4-mini | is mri recent | 92.00 | 0.80(0.33-1.00) | 0.95(0.85-1.00 | 0.80 | 0.95 | 0.80(0.40-1.00) | 0.75(0.43-1.08) |
| OpenAI o4-mini | was pet spect completed | 96.00 | 1.00(1.00-1.00) | 0.94(0.81-1.00 | 0.91 | 1.00 | 0.95(0.82-1.00) | 0.92(0.78-1.07) |
| OpenAI o4-mini | was neuropsychological evaluation completed | 100.00 | 1.00(1.00-1.00) | 1.00(1.00-1.00 | 1.00 | 1.00 | 1.00(1.00-1.00) | 1(1-1) |
| OpenAI o4-mini | was fmri completed | 100.00 | 1.00(1.00-1.00) | 1.00(1.00-1.00 | 1.00 | 1.00 | 1.00(1.00-1.00) | 1(1-1) |
| GPT-5 mini | was video eeg completed | 85.00 | 0.88(0.69-1.00) | 0.83(0.58-1.00 | 0.88 | 0.83 | 0.88(0.72-0.98) | 0.71(0.44-0.97) |
| GPT-5 mini | is mri recent | 92.00 | 0.80(0.33-1.00) | 0.95(0.85-1.00 | 0.80 | 0.95 | 0.80(0.44-1.00) | 0.75(0.43-1.08) |
| GPT-5 mini | was pet spect completed | 96.00 | 1.00(1.00-1.00) | 0.94(0.81-1.00 | 0.91 | 1.00 | 0.95(0.82-1.00) | 0.92(0.78-1.07) |
| GPT-5 mini | was neuropsychological evaluation completed | 100.00 | 1.00(1.00-1.00) | 1.00(1.00-1.00 | 1.00 | 1.00 | 1.00(1.00-1.00) | 1(1-1) |
| GPT-5 mini | was fmri completed | 100.00 | 1.00(1.00-1.00) | 1.00(1.00-1.00 | 1.00 | 1.00 | 1.00(1.00-1.00) | 1(1-1) |
| GPT-5 | was video eeg completed | 92.00 | 0.88(0.69-1.00) | 1.00(1.00-1.00 | 1.00 | 0.86 | 0.93(0.82-1.00) | 0.86(0.67-1.05) |
| GPT-5 | is mri recent | 92.00 | 0.80(0.33-1.00) | 0.95(0.85-1.00 | 0.80 | 0.95 | 0.80(0.40-1.00) | 0.75(0.43-1.08) |
| GPT-5 | was pet spect completed | 96.00 | 1.00(1.00-1.00) | 0.94(0.81-1.00 | 0.91 | 1.00 | 0.95(0.82-1.00) | 0.92(0.78-1.07) |
| GPT-5 | was neuropsychological evaluation completed | 100.00 | 1.00(1.00-1.00) | 1.00(1.00-1.00 | 1.00 | 1.00 | 1.00(1.00-1.00) | 1(1-1) |
| GPT-5 | was fmri completed | 100.00 | 1.00(1.00-1.00) | 1.00(1.00-1.00 | 1.00 | 1.00 | 1.00(1.00-1.00) | 1(1-1) |
| Majority Vote | was video eeg completed | 89.00 | 0.88(0.69-1.00) | 0.92(0.71-1.00 | 0.93 | 0.85 | 0.90(0.76-1.00) | 0.78(0.55-1.01) |
| Majority Vote | is mri recent | 92.00 | 0.80(0.33-1.00) | 0.95(0.85-1.00 | 0.80 | 0.95 | 0.80(0.40-1.00) | 0.75(0.43-1.08) |
| Majority Vote | was pet spect completed | 96.00 | 1.00(1.00-1.00) | 0.94(0.81-1.00 | 0.91 | 1.00 | 0.95(0.82-1.00) | 0.92(0.78-1.07) |
| Majority Vote | was neuropsychological evaluation completed | 100.00 | 1.00(1.00-1.00) | 1.00(1.00-1.00 | 1.00 | 1.00 | 1.00(1.00-1.00) | 1(1-1) |
| Majority Vote | was fmri completed | 100.00 | 1.00(1.00-1.00) | 1.00(1.00-1.00 | 1.00 | 1.00 | 1.00(1.00-1.00) | 1(1-1) |

Supplementary Table 4 - Assessment of Whether Surgical Option Was Already Discussed by Model

| **model** | **category** | **agreement (%)** | **sensitivity (95% CI)** | **specificity (95% CI)** | **ppv** | **npv** | **F1 (95% CI)** | **kappa** |
| --- | --- | --- | --- | --- | --- | --- | --- | --- |
| Gemini 2.0 Flash | is_after_epilepsy_surgery | 98.00 | 1.00(1.00-1.00) | 0.98(0.95-1.00) | 0.88 | 1.00 | 0.93(0.73-1.00) | 0.93 |
| Gemini 2.0 Flash | surgery_already_considered | 87.00 | 0.91(0.78-1.00) | 0.86(0.77-0.93) | 0.68 | 0.97 | 0.78(0.64-0.89) | 0.69 |
| Gemini 2.0 Flash | surgery_rejected_by_patient | 88.00 | 0.60(0.00-1.00) | 0.95(0.85-1.00) | 0.75 | 0.91 | 0.67(0.29-1.00) | 0.60 |
| Gemini 2.5 Flash | is_after_epilepsy_surgery | 100.00 | 1.00(1.00-1.00) | 1.00(1.00-1.00) | 1.00 | 1.00 | 1.00(1.00-1.00) | 1.00 |
| Gemini 2.5 Flash | surgery_already_considered | 92.00 | 1.00(1.00-1.00) | 0.90(0.83-0.97) | 0.77 | 1.00 | 0.87(0.75-0.95) | 0.82 |
| Gemini 2.5 Flash | surgery_rejected_by_patient | 100.00 | 1.00(1.00-1.00) | 1.00(1.00-1.00) | 1.00 | 1.00 | 1.00(1.00-1.00) | 1.00 |
| Gemini 2.5 Pro | is_after_epilepsy_surgery | 100.00 | 1.00(1.00-1.00) | 1.00(1.00-1.00) | 1.00 | 1.00 | 1.00(1.00-1.00) | 1.00 |
| Gemini 2.5 Pro | surgery_already_considered | 88.00 | 0.91(0.78-1.00) | 0.87(0.79-0.94) | 0.70 | 0.97 | 0.79(0.65-0.90) | 0.71 |
| Gemini 2.5 Pro | surgery_rejected_by_patient | 85.00 | 1.00(1.00-1.00) | 0.82(0.64-0.96) | 0.56 | 1.00 | 0.71(0.36-0.94) | 0.63 |
| OpenAI o4-mini | is_after_epilepsy_surgery | 100.00 | 1.00(1.00-1.00) | 1.00(1.00-1.00) | 1.00 | 1.00 | 1.00(1.00-1.00) | 1.00 |
| OpenAI o4-mini | surgery_already_considered | 90.00 | 0.91(0.77-1.00) | 0.90(0.83-0.96) | 0.75 | 0.97 | 0.82(0.69-0.92) | 0.76 |
| OpenAI o4-mini | surgery_rejected_by_patient | 96.00 | 1.00(1.00-1.00) | 0.95(0.85-1.00) | 0.83 | 1.00 | 0.91(0.67-1.00) | 0.89 |
| GPT-5 mini | is_after_epilepsy_surgery | 100.00 | 1.00(1.00-1.00) | 1.00(1.00-1.00) | 1.00 | 1.00 | 1.00(1.00-1.00) | 1.00 |
| GPT-5 mini | surgery_already_considered | 91.00 | 0.96(0.86-1.00) | 0.90(0.83-0.96) | 0.76 | 0.98 | 0.85(0.73-0.94) | 0.79 |
| GPT-5 mini | surgery_rejected_by_patient | 100.00 | 1.00(1.00-1.00) | 1.00(1.00-1.00) | 1.00 | 1.00 | 1.00(1.00-1.00) | 1.00 |
| GPT-5 | is_after_epilepsy_surgery | 100.00 | 1.00(1.00-1.00) | 1.00(1.00-1.00) | 1.00 | 1.00 | 1.00(1.00-1.00) | 1.00 |
| GPT-5 | surgery_already_considered | 92.00 | 0.96(0.85-1.00) | 0.92(0.85-0.97) | 0.79 | 0.98 | 0.86(0.74-0.95) | 0.81 |
| GPT-5 | surgery_rejected_by_patient | 100.00 | 1.00(1.00-1.00) | 1.00(1.00-1.00) | 1.00 | 1.00 | 1.00(1.00-1.00) | 1.00 |
| Majority Vote | is_after_epilepsy_surgery | 100.00 | 1.00(1.00-1.00) | 1.00(1.00-1.00) | 1.00 | 1.00 | 1.00(1.00-1.00) | 1.00 |
| Majority Vote | surgery_already_considered | 90.00 | 0.91(0.78-1.00) | 0.90(0.83-0.96) | 0.75 | 0.97 | 0.82(0.69-0.93) | 0.76 |
| Majority Vote | surgery_rejected_by_patient | 100.00 | 1.00(1.00-1.00) | 1.00(1.00-1.00) | 1.00 | 1.00 | 1.00(1.00-1.00) | 1.00 |
